# A case report of CLIPPERS syndrome and literature review

**DOI:** 10.1097/MD.0000000000026090

**Published:** 2021-06-04

**Authors:** Lei Zhang, Dan Zhao, Jia Li, Jingqi Feng, Qinghui Zhang, Li Liu, Jingxin Liu, Jiajun Chen

**Affiliations:** a Department of Neurology, China-Japan Union Hospital of Jilin University, Changchun, Jilin; b Department of Imaging Medicine, China-Japan Union Hospital of Jilin University, Changchun, China.

**Keywords:** chronic lymphocytic inflammation with pontine perivascular enhancement responsive to steroids, encephalitis, next-generation sequencing

## Abstract

**Rationale:**

Chronic lymphocytic inflammation with pontine perivascular enhancement responsive to steroids (CLIPPERS) is a chronic inflammatory disorder of the central nervous system. It is characterized by the appearance on magnetic resonance imaging of punctate and curvilinear gadolinium enhancement in the pons and cerebellum, and is exquisitely responsive to steroid treatment. The etiology of CLIPPERS remains unclear, although its pathogenesis reflects immune-mediated processes. The accurate diagnosis of this disease is very important for both its management and prognosis.

**Patient concerns:**

A 43-year-old woman presented with clinical and radiological features suggestive of CLIPPERS. Whole-exome sequencing of the patient's DNA revealed 76 mutations.

**Diagnoses:**

The patient was clinically diagnosed with CLIPPERS.

**Interventions:**

Hormone therapy was administered intravenously upon hospitalization and then gradually reduced to an oral dose.

**Outcomes:**

The clinical symptoms and imaging manifestations of the patient improved rapidly. This patient was followed up for more than 1 year, and there has been no recurrence or aggravation.

**Lessons:**

A gene variation library of CLIPPERS syndrome was established, which lays the foundation for the further accumulation of data, and will allow the etiology and pathogenesis of the disease to be explored.

## 1 Introduction

Chronic lymphocytic inflammation with pontine perivascular enhancement responsive to steroids (CLIPPERS) syndrome is a chronic inflammatory disease of the central nervous system. It mainly involves the perivascular areas of the pons, midbrain, and cerebellum, which are infiltrated by lymphocytes. Its clinical presentation varies with the location of the lesion, but predominantly includes gait ataxia and dysarthria. Head magnetic resonance imaging (MRI) shows typical pepper-like speckles, with curvilinear enhancement lesions involving the brainstem, cerebellum, and spinal cord. CLIPPERS can be effectively treated with steroid hormones. It was first reported by Pittock in 2010,^[1]^ and to date, only about 100 cases have been reported. Because of its relatively recent discovery and rare pathological data, all reported cases have been sporadic. Therefore, the etiology and pathogenesis of this disease remain unclear. Here, we report a case of clinically diagnosed CLIPPERS syndrome. Whole-exome sequencing of the patient's DNA was performed, and 76 mutations were detected. A gene variation library of CLIPPERS syndrome was thus established for the first time, which lays the foundation for the further accumulation of data, and will allow the etiology and pathogenesis of the disease to be explored.

## 2 Clinical data

The patient, a 43-year-old woman, was admitted to the hospital on January 6, 2019. Three months before hospitalization, the patient presented with paresthesia in both lower limbs and double vision without any obvious cause. The paresthesia manifested as an inability to consciously feel the existence of both of her lower limbs; however, the sensations of touch, pain, cold, and heat were all normal. She consciously felt that it was difficult to walk, but others observed that her walking posture and gait were the same as before the disease onset. Her double vision was most obvious when her eyes gazed horizontally to the left. The disease was most severe during the day, after work, and when the patient was fatigued. However, its severity was relieved slightly after resting at night. The symptoms did not affect the patient's daily life. One month before hospitalization, without any obvious inducement, the patient consciously felt unsteady after getting out of bed, and needed help to stand steadily and maintain her balance. After walking for a certain distance, she said she was able to continue walking only after taking a rest. The patient had previously been healthy.

This study was approved by the Ethics Committee of the China-Japan Union Hospital of Jilin University. The patient and her family have provided written informed consent to the participation in the study and authorized to publish the study in accordance with the Declaration of Helsinki.

### 2.1 Physical examination

The patient's general condition was good. Nervous system examination revealed that her pupils were equal in size and diameter, and were sensitive to light. Bilateral frontal lines were symmetrical, tongue extension was centered, and her left nasolabial fold was shallow. Bilateral pharyngeal reflexes existed. Muscle strength of the limbs was level 5 and muscle tension of the limbs was normal. Tendon reflexes were present in the limbs. The bilateral finger–nose test, rotation test, and heel–knee–tibia test results were unstable. The patient had bilateral pathology signs (−), normal sensation, no stiff neck, and Klinefelter Syndrome (−).

### 2.2 Laboratory investigation

After hospitalization, the patient's blood routine, urine routine, blood biochemistry, coagulation routine, immune routine, tumor markers, anti-nuclear antibody, anti-neutrophil cytoplasmic antibody, lymphocyte immunoassay, blood homocysteine, and other indicators were all normal. Fasting blood glucose was 7.27 mmol/L. No abnormalities were identified in the transcranial doppler ultrasound or electroencephalogram of the cervical vessels.

### 2.3 Magnetic resonance imaging

**Figure 1:**
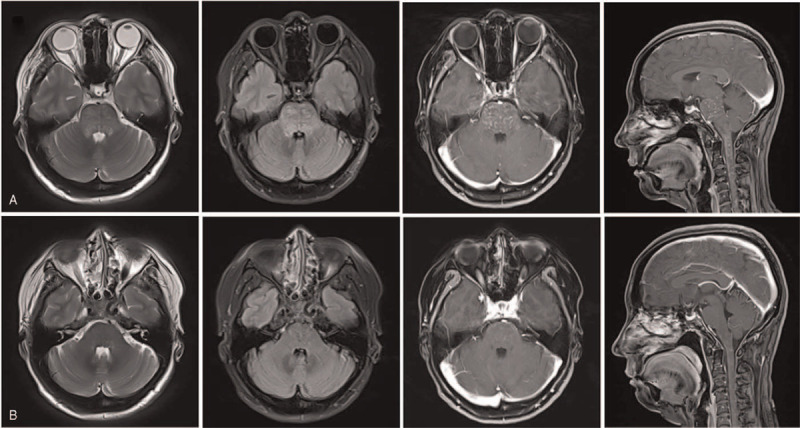
Representative T2, FLAIR, and T1 post-gadolinium MRI images of the patient. (A) T2, FLAIR, and T1 post-gadolinium MRI images of the patient before treatment. (B) T2, FLAIR, and T1 post-gadolinium MRI images of the patient after treatment. MRI = magnetic resonance imaging.

The patient underwent her first brain MRI before hospitalization (October 10, 2018). The brainstem, bilateral cerebellar hemispheres, and cervical spinal cord had scattered spots and patches, with T1 slightly longer than the T2 signal. The boundary was fuzzy. For fluid-attenuated inversion recovery (FLAIR), the signal was slightly higher, and there was no obvious enhancement on the enhanced scan. (Fig. 1A) No clear abnormalities were observed in the head magnetic resonance perfusion. The cervical and thoracic medullary in the plain and enhanced scans indicated an uneven intramedullary signal. Patches and strips were observed, with T1 slightly longer than the T2 signal. The boundary was fuzzy and the pressure lipid sequence presented high signal. The enhanced scan showed clear “pepper-like” speckled enhancement (Fig. 1A).

### 2.4 Treatment

Hormone therapy was administered upon hospitalization; 500 mg of methylprednisolone was administered intravenously once daily for 5 days, and then gradually reduced to an oral dose. The patient was discharged after her condition stabilized and her symptoms improved. Three months after treatment, the patient came in for a re-examination, and her clinical symptoms of bilateral lower limb paresthesia and double vision had disappeared completely, without discomfort. MRI was performed again. The abnormal signal shadows of multiple spots and patches in the brainstem, bilateral cerebellar hemisphere, cervical spinal cord, and thoracic spinal cord had almost completely disappeared in the plain scan sequence (Fig. 1B). This patient was followed up for more than 1 year, and there has been no recurrence or aggravation.

### 2.5 Genetic testing

During hospitalization, 2 mL of peripheral venous blood was collected from the patient in an ethylenediaminetetraacetic acid (EDTA) anticoagulant tube, and peripheral blood genomic DNA was extracted using a genomic DNA extraction kit. The obtained concentration was > 60 ng/μL, the total DNA content was > 2.0 μg, and D (260 nm)/D (280 nm) was 1.8 to 2.0. The samples were stored at −20°C. Genomic DNA was sequenced after ultrasonic disruption, library construction, hybridization capture, quality control, and other processes were performed. The sequencing results were compared with the University of California Santa Cruz hg19 reference genome using BWA0.6.2-r126 software. Repetitive sequences and low-quality data were removed, and GATK software was used for base quality correction and local realignment. The obtained single nucleotide variants (SNVs) and short insertions and deletions (indels) were annotated, and the sequencing depth, coverage depth, and uniformity were counted. Using dbSNP137 (http://www.bioinfo.org.cn/relative/dbSNP%20Home%20Page.htm), ExAC Browser (http://exac.broadinstitute.org/), the 1000 Genomes Project (http://www.internationalgenome.org/), the Genome Aggregation Database (http://gnomadold.broadinstitute.org/), and other databases, the minimum allele frequency of mutation was obtained. Mutation Taster (http://www.mutationtaster.org) was then used to predict harm. Multiple homologous sequence alignment was used to analyze the conservation of mutation sites in different species. Seventy-six mutations were identified (Table 1).

**Table 1:** Gene variants identified by next-generation sequencing.

No.	Chromosome	Gene	Codon_change
1	chr18	ADCYAP1	c.^∗^258_^∗^271delTATATATATATATA
2	chr12	LRP1	n.5112_5114delAAA
3	chr1	GSTM2	n.942_943insA
4	chr5	EBF1	n.601delA
5	chr19	TBXA2R	c.984-3dupT
6	chrX	SMS	c.34C>T
7	chrMT	MT-ND2	c.355A>G
8	chrMT	MT-CYB	c.20C>T
9	chrMT	MT-CYB	c.580A>G
10	chrMT	MT-ATP6	c.268C>T
11	chr11	NAP1L4	n.707G>C
12	chr15	NBEAP1	n.282+2T>C
13	chr20	SOX12	c.^∗^1665delT
14	chr11	ZNF195	c.^∗^352_^∗^353insAATCCAATTTAAGTAAACAATGGAGGATTTGAAGGAGAAAGGAATAGAGCAC
15	chr15	CA12	c.^∗^1596_^∗^1597dupAA
16	chr17	RABEP1	c.^∗^369_^∗^393delTAGTGTTTGGAATTTTCTGTTCATA
17	chr19	ZNF100	c.^∗^3917_^∗^3918insATTT
18	chr13	DGKH	c.623-12_623-3dupTTTTTTTTTT
19	chr4	PPARGC1A	n.53-7_53-5delTTT
20	chr21	USP16	c.-136-5G>T
21	chr16	MVP	c.-148dupC
22	chr2	LPIN1	n.614_^∗^40delCATCTCAAAAAAAAAAAAAAAAAAAAAGTGTGAGAGAGAGGCAGTGGGAGGCTCCC
23	chr8	NCALD	c.-240-9_-240-8delTT
24	chr14	AP4S1	c.367-5_367-4dupTT
25	chr4	RCHY1	c.^∗^711_^∗^8626insAG
26	chr8	VPS13B	c.9406-3dupT
27	chr9	ASPN	c.153_155delTGA
28	chr10	APBB1IP	c.-203+7_-203+8insA
29	chr11	ASRGL1	n.636_642delAAAAAAA
30	chr13	SLAIN1	c.220_221dupGG
31	chr1	RNF19B	c.185A>C
32	chr3	NMD3	c.-20-22_-20-6delTTTTTTTTTTTTTTTTT
33	chr5	CENPK	c.242-9_242-8dupTT
34	chr2	FBLN7	n.4597_4598delAA
35	chr17	CCDC40	n.3953_^∗^20delGAAAAGAAAAGAAAAGAAAAGAAAAGAAAA
36	chr8	HAS2-AS1	n.824-8G>T
37	chr1	DMRTA2	c.1187_1192delCCGCCG
38	chr16	NLRC3	n.2665-11_2665-8delATTT
39	chr5	MCTP1	n.443C>G
40	chr12	CASC1	c.1894-3_1894-2insTTTTTTTTTTTTTTTTTTTT
41	chr5	CTB-43E15.3	n.193-5_193-2delCACA
42	chr10	CCNJ	c.^∗^1347+2delT
43	chr12	RP11-20D14.6	n.463_487+1delTGAGACCATCACCTATAGCTGAGCGG
44	chr14	IGHJ6	c.18_19delCA
45	chr14	IGHJ6	c.15_16insGG
45	chr17	LINC00854	n.97+2T>C
47	chr19	ZNF571	c.^∗^794-15_^∗^812delACATATATTCCACATGTGTGTATGTGGAATATAT
48	chr22	FAM230C	n.239+12_250delCGAGGACGCCGCCCAGGGCATCGCCAA
49	chr1	SMIM12	c.^∗^3029_^∗^3030dupAA
50	chr1	RP11-230B22.1	n.172-7_172-5dupTTT
51	chr1	ANKRD45	n.1817_1824dupAAAAAAAA
52	chr1	RNY4P16	n.99_^∗^6delGAAGAAGAA
53	chr2	AC006994.3	n.82_96delGGCGGCGGCGGCGGC
54	chr3	THUMPD3	c.-257-8delT
55	chr3	RP11-372E1.6	n.263+8C>T
56	chr3	FAM194A	c.138_158dupAGAGGTGGAGGAGGAGGAGGA
57	chr3	RP11-298O21.6	n.448_455delGTGTGTGT
58	chr5	CTC-255N20.1	n.323-16_323-5delTTTTTTTTTTTT
59	chr7	CCT6P3	n.1303-5T>C
60	chr7	RP11-395B7.2	n.1696-7G>C
61	chr9	RP11-65J3.3	n.488C>T
62	chr11	AP003062.1	c.104C>G
63	chr12	TSPAN19	n.51-5_51-4delTT
64	chr15	RP11-566K19.5	n.228C>T
65	chr15	GOLGA6L2	c.2279A>G
66	chr16	RP11-166B2.1	n.77-6dupT
67	chr16	NPIPB5	c.1561G>C
68	chr16	RP11-55869A11.3	n.338-8delT
69	chr17	KRBA2	c.^∗^495_^∗^497delAAA
70	chr17	AC090616.2	c.265_267delCGG
71	chr19	CTB-25B13.12	n.632_^∗^1insCCCCCCCCC
72	chr19	ZNF587	n.190-13_190-5dupTTTTTTTTT
73	chrX	RP13-150K15.1	n.1751-8dupA
74	chrGL000219.1	AL592183.1	c.357A>T
75	chrGL000212.1	AL356585.1	c.40C>T
76	chrGL000212.1	AL356585.1	c.1289T>C

## 3 Discussion and literature review

CLIPPERS syndrome is a rare chronic inflammatory disease of the central nervous system that was first reported in 2010.^[1]^ The disease can occur in all age groups, and both men and women can be affected. In the cases reported to date, there have been slightly more male patients than female patients, and the disease has occurred mostly in young and middle-aged individuals, and occasionally in children. A pediatric case with 6 years of follow-up has been reported abroad.^[2]^ Patients tend to have subacute onset and progressive aggravation. The main lesions are in the pons, but CLIPPERS pathology can also involve other parts of the brainstem, corpus callosum, white matter of the cerebellar and basal ganglia areas, and spinal cord. The main clinical manifestations are ataxia, dysarthria, diplopia, and sensory disorders, and can include a combination of different symptoms according to the location of the lesion. Very few patients present with a single symptom (such as gait abnormalities, dysphagia, eye movement disorders, or facial numbness). CLIPPERS can also be accompanied by other non-specific symptoms, such as dizziness, nausea, vomiting, dysphagia, choking on water, and cognitive impairment. The signs and symptoms vary according to the location of the lesion.

At present, the etiology of CLIPPERS syndrome remains unknown. Some evidence suggests that CLIPPERS might be a pre-lymphoma state. Mele et al.^[3]^ demonstrated that CLIPPERS syndrome is an autoimmune disease that is mediated by T helper 17 cells. Its pathogenesis supports this opinion, and suggests a possible symptomatic lymphohistiocytic immune reaction.^[4]^ Histopathologically, CLIPPERS is an inflammatory lesion with the infiltration of lymphocytes around small blood vessels, involving both the white and gray matter. Meningeal inflammation can also be observed. The main lymphocytic components are CD3+ T lymphocytes, with a smaller population of CD2+ B lymphocytes; these are usually accompanied by scattered mature-appearing plasma cells. Neutrophils and eosinophils can also be present. Despite intense inflammatory infiltration among CLIPPERS cases, vasculitis, such as vessel wall splitting or prominent vascular lymphocytic infiltration, has not been observed.^[4]^

The imaging diagnosis of CLIPPERS syndrome is mainly performed using enhanced MRI head scans. The imaging manifestations are multiple points in the pons, midbrain, and cerebellum, as well as thin curvilinear high signal enhancement shadows, presenting pepper-like enhancement. These imaging details currently make up the evidence needed for a clinical diagnosis of CLIPPERS syndrome. At present, there is no unified diagnostic standard for CLIPPERS syndrome, and clinical diagnosis mainly depends on the following points:

(1) Subacute onset, progressive development, and symptoms of brainstem and cerebellar damage, such as ataxia, dysarthria, diplopia, and sensory disorders.
(2) Head MRI showing typical pepper-like speckles and curvilinear enhancement lesions involving the brainstem, cerebellum, and spinal cord.
(3) Pathological biopsy of the brain demonstrating an inflammatory response of infiltrating T lymphocytes around small blood vessels.
(4) Sensitivity to corticosteroid therapy.
(5) Exclusion of other systemic diseases.

The onset age of the current patient was 43 years old, and the clinical manifestations were paresthesia in both lower limbs and double vision, without specific indications in laboratory examinations. MRI revealed that the brainstem, cerebellum, and spinal cord were speckled with abnormal signal shadows. After enhancement, these regions presented “pepper-like” irregular reinforcement. Hormone therapy was effective. On the whole, the patient conformed to the performance of typical CLIPPERS syndrome.^[4]^

The main treatment for CLIPPERS syndrome is a large dose of corticosteroids. After treatment, the clinical symptoms and imaging manifestations of the present patient improved rapidly. Head MRI enhancements of patients with CLIPPERS syndrome before and after treatment have been compared, and indicated that the characteristic signs of pepper-like symptoms decreased or disappeared after treatment. However, no clinical studies have shown that this disease can be cured, so long-term hormone maintenance is needed to prevent disease recurrence. In addition, trials of immunoglobulin have been reported in the literature,^[5]^ but this treatment regime may not be effective, and remains to be further explored. In addition, some scholars have reported that hydroxychloroquine can be used for the treatment of CLIPPERS syndrome, and not only leads to rapid remission, but also effectively prevents recurrence with less adverse reactions.^[6]^

In the present research, whole-exome sequencing was performed on a patient with clinically diagnosed CLIPPERS syndrome, and 76 mutations in different genes were detected. The functions of these genes varied widely. For example, *ADCYAP1* encodes pituitary adenylate cyclase-activating polypeptide, which can stimulate adenylate cyclase. *LRP1* encodes low density lipoprotein receptor-related protein 1, which interacts with many secreted proteins and cell surface molecules and mediates their endocytosis, and/or activates signaling pathways through multiple cytosolic adaptor and scaffold proteins. *ZNF195* and *ZNF100* encode parts of zinc finger proteins, which bind nucleic acids and perform many key functions, the most important of which is transcription regulation. Of note, some genes may provide new clues for the etiology and pathogenesis of CLIPPERS. For example, EBF is a tissue-specific and differentiation stage-specific DNA-binding protein that participates in the regulation of the pre-B and B lymphocyte-specific *MB1* gene. In addition, the small GTPase Ras-related protein Rab-5 (RAB5) is a rate-limiting component in membrane docking or fusion in the early endocytic pathway.

In the present study, a gene variation library of CLIPPERS syndrome was established for the first time. This lays the foundation for the further accumulation of data and will allow the etiology and pathogenesis of the disease to be explored. It is hoped that the findings in this report will lead to the development of new and effective strategies for the clinical treatment of CLIPPERS syndrome.

## Author contributions

**Supervision:** Lei Zhang, Dan Zhao.

**Methodology:** Jia Li, Jingqi Feng.

**Writing – original draft:** Qinghui Zhang, Li Liu.

**Writing – review & editing:** Jingxin Liu, Jiajun Chen.
